# How likely is the patient to be in cardiac arrest? Caller breathing descriptors in ambulance calls that were dispatched as cardiac arrest

**DOI:** 10.1016/j.resplu.2025.100990

**Published:** 2025-05-23

**Authors:** Nirukshi Perera, Marine Riou, Tanya Birnie, Judith Finn, Austin Whiteside, David Majewski, Stephen Ball

**Affiliations:** a Prehospital, Resuscitation and Emergency Care Research Unit (PRECRU), School of Nursing, Curtin University, Bentley, Western Australia, Australia; b Centre de Recherche en Linguistique Appliquée (CeRLA), Université Lumière Lyon 2, France; c Institut Universitaire de France (IUF), France; d St John Western Australia, Belmont, Western Australia, Australia; e Department of Epidemiology and Preventive Medicine, Monash University, Victoria, Australia; f Medical School (Emergency Medicine), The University of Western Australia, Crawley, Western Australia, Australia

**Keywords:** Out-of-hospital cardiac arrest, Agonal breathing, Emergency medical services, Call-taker, Recognition, Emergency medical dispatch, Communication

## Abstract

**Background:**

In emergency ambulance calls, callers use a variety of ways to describe patients’ breathing, or the absence thereof. Call-takers have the task of interpreting these descriptions, and for unconscious patients, determining if they fit with the dispatch system’s requirements for a cardiac arrest. We aimed to categorise the breathing descriptions callers use and determine the likelihood of patients being in cardiac arrest for different breathing description categories.

**Method:**

Using call audio and ambulance dispatch data from St John Western Australia (Jan-Jun 2021) for cases dispatched as out-of-hospital cardiac arrest (OHCA) during initial “case entry” questioning, we created a schema of breathing descriptors and coded calls for their occurrence. For each descriptor we determined the percentage of cases confirmed by Emergency Medical Services (EMS) as being in arrest (true positive cases) on arrival at the scene.

**Results:**

Of 375 cases dispatched as OHCA, 85.3% (320) were true positives. Callers used a wide range of descriptors, across 23 categories. Descriptors with a high percentage of true positive cases were *Dead*, *NOT breathing, Blue/Purple* and *Unsure.* Some descriptors, notably *Barely*, *Gasp* and *Laboured* were less commonly OHCA, but still had over 50% true positives.

**Conclusion:**

Patients who are dispatched as OHCA by call-takers have a diverse range of caller descriptors for their breathing status. While descriptor categories varied in the percentage of EMS-confirmed OHCAs, none had a low percentage. We recommend exposing call-takers to the broad range of breathing descriptors which can be applied to their role in identifying OHCA and addressing caller perceptions of patient signs of life.

## Highlights

• Callers use a wide variety of descriptors for unconscious patients’ breathing.
• Descriptors *Dead, NOT breathing, Blue/Purple, Unsure* highly associated with OHCA.
• *Gasp*, *Barely*, *Laboured* less commonly OHCA, but still over 50% true positives.
• Opportunity to educate call-takers on range of callers’ breathing descriptors.

## Introduction

In an emergency call for a patient who is having an out-of-hospital cardiac arrest (OHCA), it is important that the arrest is recognised as soon as possible by the call-taker, to enable early high-priority ambulance dispatch and provide the caller with over-the-phone instructions on cardiopulmonary resuscitation (CPR) and defibrillation.^1^, ^2^, ^3^ However, a widespread challenge for OHCA recognition is agonal breathing, which is common in the minutes after the arrest,^4^, ^5^, ^6^ and prone to misinterpretation that the patient is still breathing effectively.^7^, ^8^ Dispatch systems therefore list a number of breathing descriptions that signal cardiac arrest for unconscious patients.^9^ For example, the Medical Priority Dispatch System (MPDS) proposes that an unconscious patient should be treated as a cardiac arrest if they are described as “not breathing”, “barely breathing”, “can’t breathe at all”, “fighting for air”, “gasping for air”, [breathing] “just a little”, “making funny noises”, or “reasonable equivalents”.^10^

While call-takers are expected to initiate dispatcher-assisted CPR^11^ as soon as possible after OHCA recognition, callers sometimes resist resuscitation on the basis that they think the patient is still alive^12^ due to the presence of abnormal/agonal breathing.^13^ If call-takers are to successfully convince callers to perform CPR and defibrillation, it is important that they themselves are confident and assertive about treating the patient as being in arrest, even in the context of a caller’s equivocal or ambiguous descriptions of breathing.

Therefore, in this study we take a quantitative approach to informing the likelihood with which call-takers can expect that the patients they have dispatched as cardiac arrest, based on their interpretation of different caller breathing descriptions, are in fact an arrest (i.e., true positive cases). Specifically, our aims were: (1) to categorise the various breathing descriptors used by callers during case entry, and (2) to quantify the proportion of patients in cardiac arrest (i.e., positive predictive value) for different breathing descriptors.

## Methods

### Study design

We conducted a retrospective cohort study of emergency ambulance telephone calls for cases dispatched as OHCA by a single emergency medical service (EMS) over a six-month period, 1 January to 30 June 2021. We listened to call recordings and, based on our own coding schema, recorded the breathing descriptors uttered during the preliminary phase of the call (case entry).

To pursue a relatively consistent cohort, we only included patients whose arrest presented as having a non-specific cause − i.e. simply unconscious and not breathing effectively or at all. For this purpose, we only included cases that the call-taker had dispatched with an MPDS case-entry code of 09E01 (OHCA, No breathing at all) and 09E02 (OHCA, Uncertain breathing). We purposefully excluded MPDS codes that related to cardiac arrest where a particular cause was evident (e.g., allergies, chemical/fume inhalation, hanging, strangulation, suffocation). In addition, we excluded cases where the caller was not with the patient (i.e. not “second party”), and where the patient was described as awake/conscious or unequivocally alive (talking, responding or standing) during case entry. Note that, since we collected cases that were dispatched as cardiac arrest, these included a potential mix of true positive and false positive OHCA cases (which would be determined when the EMS arrived and treated the patient – see data collection section below).

Approval was obtained from the St John WA Research Governance Committee and Curtin University Human Research Ethics Committee (HR128/2013).

### Context

St John WA (SJ-WA) is the sole provider of emergency ambulance services in the State of Western Australia (WA). SJ-WA manages all WA Triple Zero (000) ambulance calls at the State Operations Centre, Perth, using the Medical Priority Dispatch System™ (MPDS) version 13.2 call-taking protocol.^10^ Further context is provided in Table 1.

**Table 1:** Characteristics of St John Western Australia call centre.

Characteristic	Detail
Number of call-takers	85
Number of calls received 1 Jan – 30 Jun 2021	110,401
Call-taker training	Clinical qualifications not required. Initial training includes: • Senior first aid qualification and medical terminology • Advanced Emergency Medical Dispatcher qualification from the International Academies of Emergency Dispatch in order to use the Medical Priority Dispatch System (MPDS) software, ProQA In-house training includes: • Caller control • Computer Aided Dispatch (CAD) system • Mapping • Wellbeing and support • Diversity and cultural awareness • Supported emergency call-taking

### Data collection

**Fig. 1:**
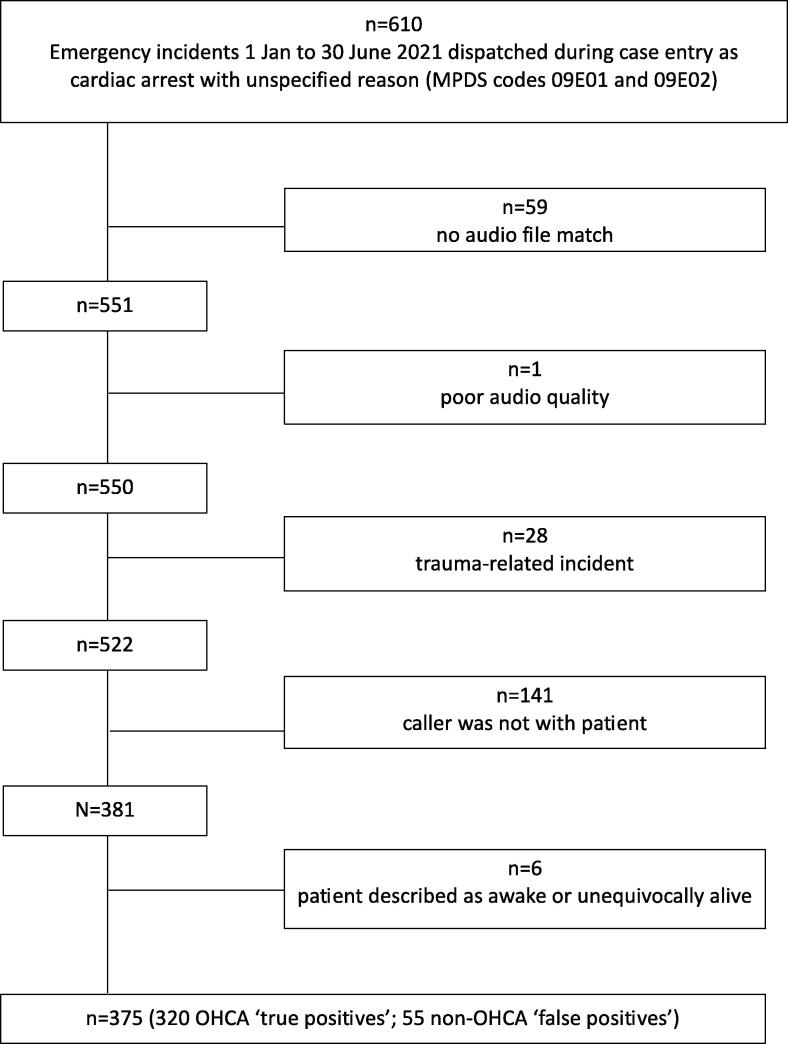
Study flow diagram. ‘True positives’ refer to cases of unwitnessed or bystander-witnessed cases of EMS-confirmed OHCA, and ‘false positives’ refers to all other cases (either EMS-witnessed cardiac arrests, or not EMS-confirmed OHCAs).

The cohort selection (Fig. 1) was based on patients dispatched by call-takers as cardiac arrest by the end of the case entry phase. As Fig. 1 shows, the extraction of audio calls returned a 90.3% match, with 9.7% (*n* = 59) of calls not returned due to limitations in our extraction syntax, which was based on searching for a unique match in call timing.

We sourced SJ-WA’s ‘ProQA’^14^ (ambulance dispatch data) records to determine which cases were dispatched as OHCA during case entry. We obtained additional data fields from the SJ-WA OHCA Registry, which records all ambulance-attended OHCAs in WA. The true positive subgroup of our cohort consisted of cases where the patient had an EMS-confirmed bystander-witnessed or unwitnessed arrest. The comparator false positive subgroup contained patients who did not arrest or arrested after ambulance arrival (EMS-witnessed OHCAs), despite having been dispatched as an OHCA. Call audio files were downloaded from SJ-WA’s call database.

### Development of coding schema

We initially listened to the first 50 audio calls (out of the study cohort of 375 calls) to compile a list of callers’ descriptors for patients’ breathing. We then designed a coding schema to classify the descriptors, in which callers’ descriptions were grouped together based on the type of meaning they conveyed, referring to the work of Fukushima et al.^15^ The investigative group, comprising of linguists, prehospital researchers, call centre management and clinicians, then refined the (non-exhaustive) list in Table 2. To determine how descriptors were grouped together, we checked their semantic proximity using WordNet®,^16^ a lexical database of English, which links words into networks of semantically related concepts.

**Table 2:** Coding schema for breathing descriptors and patient descriptors in Emergency Medical Service calls dispatched as cardiac arrest.

Category	Example words/statements (non-exhaustive)
**BREATHING STATUS CONFIRMATION**	
**YES breathing**	yeah, s/he’s breathing, I think so, looks like s/he’s breathing, seems to be
**NOT breathing**	no, nope, don’t think s/he’s breathing, I don’t believe so, doesn’t look like, doesn’t seem to be, not taking a breath, (may have) stopped breathing
**DESCRIBING BREATHING**	
**Barely**	barely, hardly, not properly, not (very) well, not (that) good/great, not really, not exactly, sort of, kind of, poorly
**Breathless**	breathless, short of breath, out of breath
**Can’t breathe**	s/he can’t breathe
**Deep**	deep, big breaths
**Erratic**	erratic, sporadic, on and off, irregular
**Laboured**	laboured, strained, heavy, struggling, challenging, trouble, trying hard, difficulty, fighting for air
**Shallow**	shallow, little/small/tiny breaths, faint, weak, feeble, (very) light(ly), (only) (just) a little (bit), slightly
**Slow**	slow
**DESCRIBING SOUND**	
**Gasp**	gasp, gasping, gulping
**Gurgle**	gurgle, gargle, sputter, splutter
**Pant**	pant, panting
**Raspy**	raspy
**Snore**	snoring
**Snort**	snort, grunt
**Strange noises**	strange/weird/odd/funny noises/sounds
**OTHER**	
**Blue/purple**	lips are blue, blue in the face, turning purple
**CPR already in progress**	we’ve started CPR/compressions
**Dead**	dead, gone, passed away, deceased
**Unsure**	unsure, not sure, uncertain, not certain, don’t know, can’t tell
**Verification strategies**	*example actions*: caller tells call-taker to listen to patient, call-taker mentions hearing agonal breathing
**Miscellaneous**	breathing problems, coughing, hiccupping, their chest/stomach is moving up and down, heave, (dry) retching

### Data analysis

**Fig. 2:**
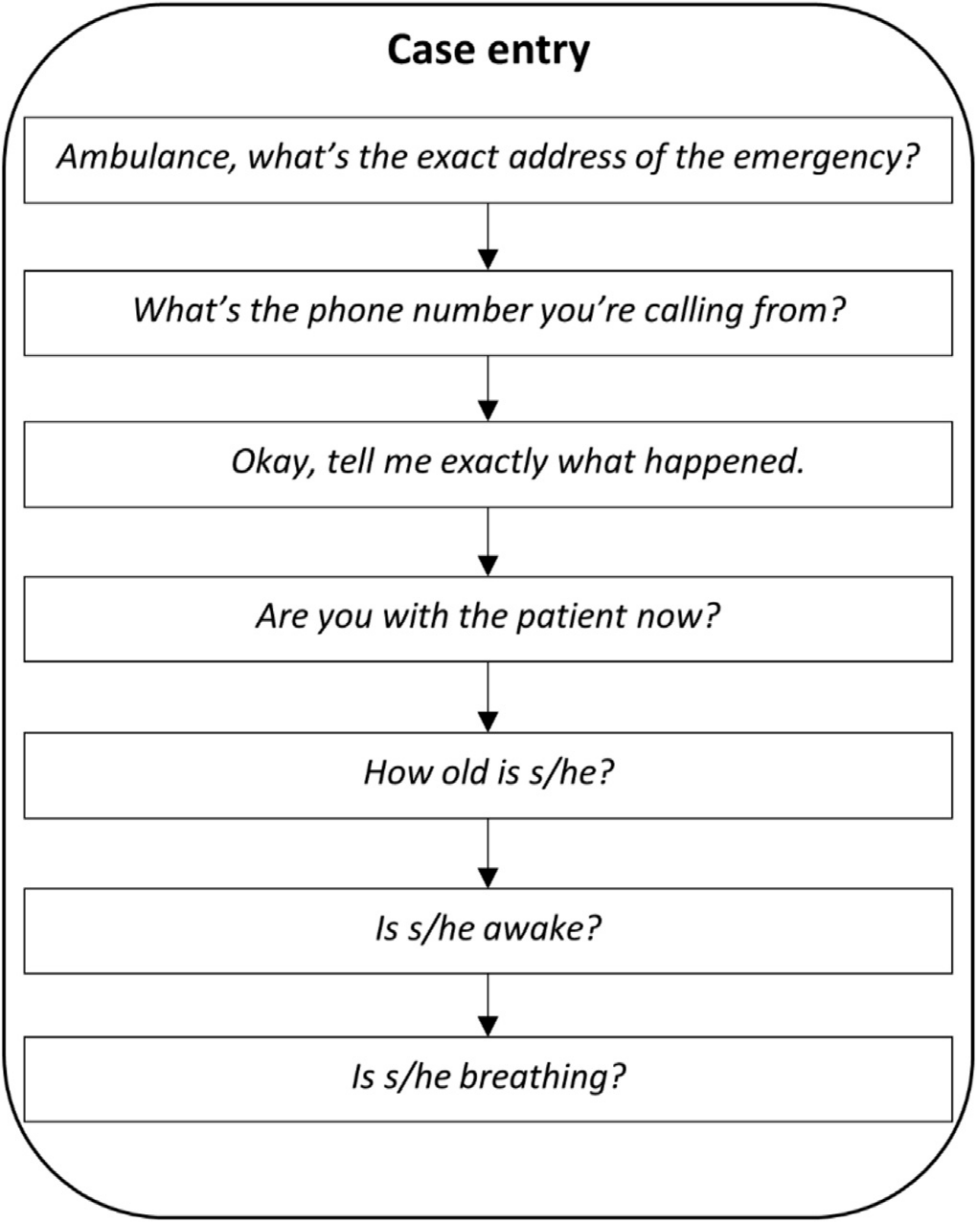
The Medical Priority Dispatch System™^10^ protocol for case entry.

We listened to call recordings and, based on the coding schema, recorded the breathing descriptors that callers uttered during the preliminary phase of the call known as “case entry” i.e., the initial screening phase of the MPDS protocol where address, phone number and awake and breathing questions are asked (Fig. 2). During this phase of the call, we recorded any caller utterances pertaining to breathing, whether it was in answer to one of the scripted prompts in Fig. 2, a call-taker check for clarification (e.g. “you said he’s breathing?”) or unsolicited by the call-taker. The end of case entry was signalled by the call-taker making some statement/question that was outside the case entry section of the MPDS protocol.

In order to represent “call-taker reality” investigators were blinded to the true/false positive status of each case, with the order of calls randomised. Two authors (TB, MR) undertook independent coding for the first 100 calls to apply the schema. Disagreements were discussed and resolved either through consensus or a third author, and the schema was updated. The remaining calls were coded independently by two authors (TB, NP) and disagreements resolved in the same way. The schema was adapted whenever coders encountered new recurring descriptors, and coding was updated accordingly. While the primary purpose of the schema was to classify breathing descriptions, other patient descriptors (e.g., the patient being described as blue/purple, or dead) were included as potentially prompting OHCA recognition. In developing the schema, it was evident that these additional descriptors were often provided as answers to the question, “Is s/he breathing?”.

After coding, we unblinded the dataset and calculated the positive predictive value, i.e., percentage of true positive EMS-confirmed (unwitnessed or bystander-witnessed) OHCAs among cases dispatched as OHCA, for each type of breathing descriptor.

## Results

The final study cohort comprised 375 emergency calls dispatched as an OHCA during case entry: 320 EMS-confirmed OHCA (true positive) cases and 55 non-OHCA (false positive but unconscious) patients (Fig. 1). Our study generated a list of 23 descriptor categories including two that confirmed the patient’s breathing status (*NOT breathing*; *YES breathing*), eight different breathing qualifications, seven descriptors of the sound of patient breathing and six ‘other’ patient descriptors (Table 2). Table 3 presents the frequency of specific breathing descriptors used in emergency calls and their association with true positive cases of OHCA.

**Table 3:** Comparison between breathing descriptors uttered in case entry, in terms of positive predictive value – i.e. percentage of EMS-confirmed cardiac arrests.

Category	Breathing descriptors	Total cases dispatched as OHCA	True positive OHCA cases	Positive predictive value (% true positive OHCA) with 95% conf. interval	
**Confirmation**	YES breathing	32	22	68.8	(50.0–83.9)
	NOT breathing	265	230	86.8	(82.1–90.6)
**Describing breathing**	Barely	11	7	63.6	(30.8–89.1)
	Breathless	1	1	100.0	(2.5–100.0)
	Can't breathe	8	6	75.0	(34.9–96.8)
	Deep	4	4	100.0	(39.8–100.0)
	Erratic	4	4	100.0	(39.8–100.0)
	Laboured	9	5	55.6	(21.2–86.3)
	Shallow	14	10	71.4	(41.9–91.6)
	Slow	2	2	100.0	(15.8–100.0)
**Describing sound**	Gasp	11	7	63.6	(30.8–89.1)
	Gurgle	5	5	100.0	(47.8–100.0)
	Pant	1	1	100.0	(2.5–100.0)
	Raspy	1	1	100.0	(2.5–100.0)
	Snore	6	5	83.3	(35.9–99.6)
	Snort	2	2	100.0	(15.8–100.0)
	Strange noises	9	7	77.8	(40.0–97.2)
**Other**	Blue/Purple	23	19	82.6	(61.2–95.0)
	CPR already in progress	14	14	100.0	(76.8–100.0)
	Dead	92	90	97.8	(92.4–99.7)
	Unsure	56	43	76.8	(63.6–87.0)
	Verification strategy (Caller tells call-taker to listen to patient)	2	0	0.0	(0.0–84.2)
	TOTAL	375	320	85.3	(81.3–88.8)

Across the study cohort (of true/false positive OHCAs), the most common breathing descriptor categories were *NOT breathing* (70.7% of all calls), *Dead* (24.5%), *Unsure* (14.9%), *YES breathing* (8.5%), and *Blue/Purple* (6.1%). Of the 32 cases where the caller stated *YES breathing*, there were 15 cases (46.9%) where the caller also stated *NOT breathing*, at some point during case entry, and 11 cases (34.4%) where *YES breathing* was accompanied by another descriptor.

Overall, 85.3% of the cases (320/375) were true positives (i.e. EMS-confirmed OHCA cases) – i.e., there was a positive predictive value of 0.85 for cases dispatched as OHCA. The more frequently occurring descriptors that displayed a high positive predictive value were *Dead* (97.8% of all instances of the *dead* descriptor category were true positives), *NOT breathing* (86.8%), *Blue/Purple* (82.6%) and *Unsure* (76.8%).

Some other descriptors also had a high association with EMS-confirmed OHCA but were not as frequent (<15 cases, i.e. <4% of cases): *Breathless* (*n* = 1, 100% true positive), *CPR already in progress* (*n* = 14, 100%), *Deep* (*n* = 4, 100%), *Erratic* (*n* = 4, 100%), *Gurgle* (*n* = 5, 100%), *Pant* (*n* = 1, 100%), *Raspy* (*n* = 1, 100%), *Slow* (*n* = 2, 100%), *Snort* (*n* = 2, 100%), *Snore* (*n* = 6, 83.3%), *Strange noises* (*n* = 9, 77.8%), *Can’t breathe* (*n* = 8, 75.0%) and *Shallow* (*n* = 14, 71.4%). Descriptors that were less commonly associated with EMS-confirmed OHCAs were *YES breathing* (68.8% were true positives), *Barely* (63.6%), *Gasp* (63.6%) and *Laboured* (55.6%).

## Discussion

This study revealed the variety of caller descriptors for patient breathing status among cases dispatched as OHCA and examined the likelihood (positive predictive value) of patients being an EMS-confirmed cardiac arrest for each breathing descriptors category.

Our results highlight the diverse ways callers describe the breathing status of unconscious patients. While some descriptors were very common, notably *Not breathing, Dead, Unsure,* and *Blue/Purple*, our results also highlight a much wider range of descriptors being used – across eight categories of breathing type and seven for breathing sound. Significantly, each descriptor category is itself an aggregate of semantically similar descriptions. For example, the category *Laboured* may also present as “strained”, “heavy”, “struggling”, “challenging”, “trouble”, “trying hard”, “difficulty” and “fighting for air”. This is a reminder that call-takers not only need to listen out for particular words but for the meaning which can be expressed in diverse ways. In this sense, our schema in Table 2 is non-exhaustive in that it can be added to and adapted to other languages and settings.

In comparing the positive predictive value of different breathing descriptors, a key finding from our study was that patients were more likely than not to be in cardiac arrest across the range of descriptors callers use (i.e. more than 50% of cases with each descriptor were true positives), with the exception of one rare category that had only two cases (*verification strategy*). While the positive predictive values often had broad confidence intervals, and therefore cannot be said to be strong indicators of patients being in cardiac arrest, our results are consistent with the precautionary approach of EMS call-takers treating non-normal breathing for unconscious patients as a presumption of cardiac arrest until proven otherwise (e.g. if the patient was to react in response to CPR). This result could be used to reassure call-takers to remain assertive in relation to OHCA recognition and the actions that follow it. For example, having confidence in OHCA recognition may be useful for call-takers in those instances where callers resist CPR instructions on the basis that patients are still showing signs of life. In this context, call-taker confidence can be supported by the evidence that any harm done from CPR to patients who are not in arrest is low relative to CPR’s life-saving value for patients who are in arrest.^11^, ^17^, ^18^

Our findings indicate that, despite the binary (yes/no) nature of the MPDS question, “*Is s/he breathing?*”, callers find many ways of providing equivocal answers. While on the one hand these descriptions provide an important opportunity for OHCA recognition, the diversity of descriptions presents a challenging environment for call-takers^19^ if they are to remain sensitive to the combination of unconsciousness and qualified breathing as indicating cardiac arrest. Focussing specifically on EMS-confirmed OHCAs (i.e. true positives), the range of breathing descriptors in our cohort indicates that the breathing descriptors of OHCA patients are much more varied than the examples listed in the MPDS as proposed indicators of ineffective/agonal breathing.^10^ There may therefore be benefit in exposing call-takers to a broader set of breathing descriptors used in relation to OHCA patients during emergency calls. At the same time, a complicating issue is that cardiac arrest calls make up a very small proportion of emergency ambulance calls,^20^ so call-takers may not be primed to react to every descriptor or combination of descriptors as solely indicating OHCA.

A priori, one of the motivations for our study was to identify any descriptors that have a low positive predictive value of being an EMS-confirmed cardiac arrest. That we didn’t find any breathing descriptors with low positive predictive values is therefore unexpected. Notably, it was even the case that calls with the *YES breathing* confirmation had a reasonably high positive predictive value, of 69%. This highlights the uncertainty that may be associated with this descriptor category*.* In relation to this, we noted multiple cases where the *YES breathing* confirmation was accompanied by breathing descriptors, e.g. *Barely* and *Laboured* (results not shown). This confirms our previous research that found that for unconscious patients, the response of “yes” to the breathing question*,* when accompanied by additional breathing descriptors, requires close attention as it does not necessarily indicate effective breathing.^21^

Another interesting category was the descriptor of the caller being *Unsure* about the patient’s breathing. Not only was this descriptor common in our cohort (in 15% of calls and the third most common category after *Not Breathing* and *Dead*), but it had a high positive predictive value of 77%. While the concept of being unsure has the inherent potential for interpretation as a lack of information (and therefore the absence of evidence), it is impressive that call-takers commonly categorise such calls as cardiac arrest, consistent with the MPDS’s explicit listing of uncertain breathing as a basis for OHCA recognition where the patient presents as unconscious.^10^ The high frequency of calls with *Unsure* breathing, and their high positive predictive value demonstrates the potential value of focusing on this particular descriptor in call-taker training.

### Limitations

In relation to our aim of comparing the positive predictive value of OHCA for different breathing descriptors, we restricted the cohort to cases that were classified as cardiac arrest during case entry. For future research, we propose additionally examining the presence of these descriptors in false negative cases – i.e. not dispatched as OHCA but subsequently confirmed by EMS as being in cardiac arrest. This was beyond the scope of our current study but would allow for statements on the absolute likelihood of specific breathing descriptors indicating cardiac arrest.

We took a thorough and systematic approach to creating the coding schema. However, we acknowledge that despite consensus amongst the co-authors, some categorisation of the descriptors is arguable, and overlaps between categories may exist. Furthermore, we only analysed breathing descriptors in isolation, i.e., our analysis did not quantify the co-occurrence of different breathing/patient descriptors within calls, which in combination may influence call-taker recognition. Notably, the finding that *YES breathing* had a positive predictive value of 69% points to the complexity of OHCA recognition, indicative that call-takers are responding to something else uttered by the caller that contradicted a simple affirmation of breathing. We therefore propose that the issue of multiple breathing descriptors in OHCA calls is a topic for future study, with a particular focus on contradictions around *YES breathing*. The nuances of these combinations, and the interactions involved between caller and call-taker, would be more appropriately analysed through linguistic methods.

## Conclusions

This study found that callers used a wide variety of breathing descriptors (23 categories in total) for the status of patients who were then dispatched as OHCA by call-takers. All the descriptors (when analysed in isolation) had a positive predictive value (i.e. the percentage of EMS-confirmed OHCAs) over 50%. These results highlight the value of providing call-takers with a broader range of breathing descriptors, used by callers, as part of their training. If call-takers have confidence that such descriptors can be indicative of an OHCA they could be better placed to address callers’ concerns that OHCA patients are showing signs of life.

## CRediT authorship contribution statement

**Nirukshi Perera:** Writing – original draft, Visualization, Validation, Project administration, Methodology, Investigation, Formal analysis, Conceptualization. **Marine Riou:** Writing – review & editing, Visualization, Validation, Resources, Methodology, Investigation, Formal analysis, Conceptualization. **Tanya Birnie:** Writing – review & editing, Validation, Resources, Methodology, Investigation, Formal analysis. **Judith Finn:** Writing – review & editing, Supervision, Funding acquisition, Conceptualization. **Austin Whiteside:** Writing – review & editing, Resources, Conceptualization. **David Majewski:** Writing – review & editing, Formal analysis, Data curation. **Stephen Ball:** Writing – original draft, Visualization, Validation, Supervision, Methodology, Funding acquisition, Formal analysis, Data curation, Conceptualization.

## Declaration of competing interest

The authors declare the following financial interests/personal relationships which may be considered as potential competing interests: The authors declare the following financial interests/personal relationships which may be considered as potential competing interests: AW is employed by St John Western Australia (SJ-WA); JF and SB hold adjunct appointments with SJ-WA and JF receives research project funding from SJ-WA. JF is on the Editorial board of Resuscitation.
